# Cross-Cultural Adaptation and Validation of Knee Quality of Life 26-Item Questionnaire into Spanish in Patellofemoral Pain

**DOI:** 10.3390/jcm15135102

**Published:** 2026-06-30

**Authors:** Fernando Espuny-Ruiz, Carmen Ridao-Fernandez, Rocio Aldon-Villegas, Maria-Luisa Benitez-Lugo, Gema Chamorro-Moriana

**Affiliations:** 1 Department of Physiotherapy, Faculty of Nursing, Physiotherapy and Podiatry, University of Seville, 41009 Seville, Spain; feresprui@alum.us.es (F.E.-R.); marisabeni@us.es (M.-L.B.-L.); gchamorro@us.es (G.C.-M.); 2 Research Group “Area of Physiotherapy CTS-305”, Department of Physiotherapy, University of Seville, 41009 Seville, Spain; 3 Faculty of Health and Life Sciences, University CEU Fernando III, CEU Universities, 41930 Seville, Spain

**Keywords:** diagnostic self-assessment, knee, patellofemoral pain syndrome, patient-reported outcome measures, quality of life, validation study

## Abstract

**Background/Objectives**: Patellofemoral pain (PFP) is a persistent knee disorder that impacts quality of life (QoL). Although holistic assessments are needed, no specific tools exist regarding QoL in PFP. The Knee Quality of Life 26-item questionnaire (KQoL-26), covering physical and psychosocial dimensions, could be useful but has not been adapted for Spanish speakers. This study aimed to cross-culturally adapt KQoL-26 into Spanish and assess its psychometric properties regarding PFP, including validity and reliability. **Methods**: This cross-cultural adaptation followed COSMIN recommendations. The cultural and linguistic adaptation of KQoL-26 included 104 subjects with PFP, involving 145 affected knees. Inclusion criteria: symptomatic PFP, aged between 16 and 55, and being native Spanish speakers. Validity (structural, construct, and external), reliability (internal consistency, test–retest, and agreement), discriminant ability, and feasibility were assessed. **Results**: Factor Analysis identified five dimensions. Convergent validity demonstrated a strong correlation between KQoL-26 and Knee Injury and Osteoarthritis Outcome Score for Patellofemoral pain and osteoarthritis, Kujala Score, Fulkerson Scale, and the Physical Component Summary of 12-item Short Form, while weak correlations with descriptive data supported discriminant validity. The questionnaire demonstrated high internal consistency (Cronbach’s α = 0.949) and test–retest reliability (ICC = 0.949), with adequate agreement parameters. No floor or ceiling effects were observed. Regarding feasibility, KQoL-26 was understandable and completed by all participants. **Conclusions**: The Spanish KQoL-26 is a valid, reliable, and feasible QoL assessment tool for Spanish speakers with PFP. Its use may support both clinical decision-making and research in this population group by providing a comprehensive assessment of patient-reported outcomes.

## 1. Introduction

Patellofemoral pain (PFP) is a musculoskeletal, frequently persistent [1] pain condition [2] characterized by anterior knee pain [3] aggravated by weight-bearing in knee-flexion positions, such as running [4], squatting [4], stair climbing [5], or descending [4]. A review reported an estimated 25% annual prevalence of PFP across diverse countries (USA, UK, Israel, Iran, etc.), with women being twice as likely to experience PFP as men [6].

PFP substantially impacts quality of life (QoL), limiting participation in sports or work-related activities without pain [7]. Despite evidence suggesting that QoL impairment in PFP is comparable to osteoarthritis and even greater than that reported in anterior cruciate ligament injuries [8], research has traditionally focused on physical aspects [9], with a biomechanical approach [10,11]. Current evidence supports a biopsychosocial approach [10,12], as psychosocial factors [10] such as kinesiophobia [7,13], depression [1], and catastrophizing [9] contribute to symptom severity and disability in PFP [10].

There are knee pathology-specific questionnaires that address QoL-related domains, particularly for ligament injuries [14,15,16]. However, there are currently no tools specifically designed to assess QoL in patellofemoral pathology. Commonly used instruments such as the Kujala Patellofemoral Score (KPS) [17] and the Fulkerson Knee Instability Scale (FKIS) [18] focus on physical/biomechanical aspects, while the Knee Injury and Osteoarthritis Outcome Score for Patellofemoral pain and osteoarthritis (KOOS-PF) [19] minimally addresses QoL with a single item.

The Knee Quality of Life 26-item questionnaire (KQoL-26), developed in 2008 [20], is currently the only functional assessment questionnaire for knee impairments in general that assesses knee-specific function, global functions, and QoL, including psychological, occupational, and sports-related dimensions [21]. Moreover, the methodological quality of its validation was highlighted positively according to Consensus-based Standards for the selection of health Measurement Instruments (COSMIN) [22], and it has been used in clinical trials [23,24,25,26], reviews [22,27,28,29], clinical practice guidelines [30,31], etc. Nevertheless, no Spanish cross-cultural adaptation or validation is currently available, limiting its use in international and multicenter research projects [32].

Considering all the above and the global relevance of Spanish [33], this study aims to cross-culturally adapt the KQoL-26 into Spanish and evaluate its psychometric properties, including validity and reliability, in individuals with PFP in Spain.

## 2. Materials and Methods

This research focused on the cross-cultural adaptation and validation of the KQoL-26 into Spanish, following the COSMIN reporting guideline for studies on measurement properties of patient-reported outcome measures (PROMs) [34], as well as the Guidelines for Reporting Reliability and Agreement Studies (GRRAS) [35]. We received permission to adapt the original questionnaire from Professor Andrew Garratt, the main developer of the questionnaire [20].

The process of cross-cultural adaptation encompassed both linguistic and cultural adjustments to the KQoL-26 and the assessment of its psychometric properties, including reliability and validity. A six-step process was conducted following the guidelines established by Beaton et al. [36]. (1) Two independent forward translations of the KQoL-26 from English to Spanish by two bilingual individuals who were native Spanish speakers. One translator had clinical expertise in musculoskeletal disorders, while the other specialized in English language studies and translation. (2) Synthesis of the two Spanish versions into a single version through a review by three experts in PFP, in collaboration with the translators, to reach a consensus. (3) Back-translation of the synthesized version by two native English speakers who are fluent in Spanish, one of whom was a musculoskeletal pathology specialist who was unfamiliar with the original KQoL-26. (4) Expert committee review of the back-translated version, comprising the translators and the three clinical experts. (5) Pilot testing of the Spanish version with 10 PFP patients to assess comprehension, clarity, vocabulary appropriateness, and cultural relevance. Any necessary modifications identified during the pilot testing would be introduced. (6) Validation process of the Spanish adaptation of the KQoL-26.

### 2.1. Participants and Sample

Participants were recruited through a non-random convenience sampling method from multiple sources, including social media, private clinics, sports clubs, and university outreach channels. Participants contacted the study team voluntarily and were scheduled consecutively upon request; participation was voluntary, and no compensation was provided. The inclusion criteria encompassed individuals experiencing symptomatic PFP, with or without cartilage involvement, which could be unilateral or bilateral. The condition referred to the patellofemoral joint conflict caused by non-traumatic factors, such as muscle imbalances, patellar maltracking, or motor control impairment. Eligible participants were aged between 16 and 55, in order to exclude late symptoms of apophysitis (e.g., Osgood–Schlatter or Sinding–Larssen–Johansson) and early osteoarthritis [20], and they were required to be native Spanish speakers. Their PFP diagnosis had either already been established by a clinician or was confirmed by the research team based on established clinical criteria, defined as pain around or behind the patella aggravated by at least one patellofemoral loading activity in a flexed knee (e.g., squatting, stair climbing, or jumping) [37]. Exclusion criteria included severe cognitive or coordination impairments, or knee dysfunctions that are unrelated to PFP, such as femoral condyle or patella fractures, prosthetic joints, neurodegenerative pathologies, tumor processes, or disorders in other joints like the femorotibial joint, hip, or ankle that might interfere with the physical assessments or skew the results, as well as ongoing rehabilitation treatment.

### 2.2. Functional Assessment Tools Used

Five self-administered questionnaires were used: one for the adaptation to Spanish (KQoL-26) [20] and four that had already been adapted for the validation process (KOOS-PF [38], KPS [39], and FKIS [40], specific for patellofemoral pathology, and the 12-Item Short Form health survey (SF-12) [41], a health-related QoL tool).

KQoL-26 [20] is a 26-item questionnaire designed in 2008 to evaluate knee-related QoL in subjects with knee injuries. Responses are scored using a five-point Likert scale, contributing to three subscales (Physical Functioning, Activity Limitations, and Emotional Functioning) where higher scores (range: 0–100) indicate better knee-related QoL.

KOOS-PF [19], 2018, consists of 11 questions with 5 possible answers scored using a Likert scale (0—none/never to 4—extreme/always) that addresses stiffness (1 item), pain (9 items), and QoL (1 item). It uses a formula, as do all Knee Injury and Osteoarthritis Outcome Score (KOOS) questionnaires [42], which converts the obtained score into a scale ranging from 0 to 100 (ideal condition).

KPS [17], 1993, comprises 13 items covering pain and physical changes (3 items), potential functional limitations (8 items), and challenges in sports participation (2 items). Response options vary from 3 to 5 per item and are scored from 0 to 10, except for some items rated from 0 to 5. The total score range is 0–100, with 100 indicating optimal condition.

FKIS [18], 1990, derived from the Lysholm Knee Score [43], comprises 7 items that assess limp, need for support, stair climbing, squatting, instability, pain, and swelling, with scores ranging 0–100 points, where 100 indicates the best condition.

SF-12 [44], 1996, includes 12 items derived from SF-36 [45]. These items address physical functioning, role limitations, bodily pain, general health perceptions, vitality, social functioning, and mental health. Responses are scored using a norm-based algorithm to generate two composite scores, the Physical Component Summary (SF-12-PCS) and the Mental Component Summary (SF-12-MCS), each ranging 0–100 points (100 indicates optimal condition).

### 2.3. Action Protocol

After the linguistic adaptation process, assessments were conducted by two experienced researchers in knee PROMs (FE and CR) between 2022 and 2024 in Seville, Spain. Each participant completed two assessment sessions spaced between 7 and 14 days apart, ensuring sufficient time to minimize recall bias while avoiding potential clinical changes in PFP [38,39], as the study was not designed to assess intervention effects. Participants experiencing substantial symptom or functional changes between sessions, such as a new injury, infiltrations, surgery, or similar events, were excluded. Descriptive data were collected, including age, weight, height, region, occupation, impairment characteristics (unilateral or bilateral, dominant or non-dominant limb), time with pain, physical activity levels, and Q-angle, after receiving detailed study information, answering questions, and signing informed consent forms. One of the researchers (FE) ensured all participants completed the assessments consistently. Participants completed the KOOS-PF, KPS, FKIS, KQoL-26, and SF-12 questionnaires in the same order during both sessions. Those with bilateral PFP provided responses for each knee individually where relevant, and participants could request clarification throughout the process. Completion time was recorded, excluding time for queries.

### 2.4. Analysis of Psychometric Properties

The psychometric properties that were assessed were structural validity, construct validity (convergent and discriminant validity), external validity, internal consistency, test–retest reliability, agreement, discriminant ability (floor and ceiling effects), and feasibility. Following the COSMIN guidelines [46,47,48], including its latest 2024 update [49], criterion validity was not analyzed, as PROMs studies inherently lack gold standards due to their subjective nature.

Structural validity analyzed the factor structure of the questionnaire to ensure that the items were appropriately grouped into dimensions.

Construct validity was analyzed to confirm the coherence of the assessed construct. Based on previous findings [20,50,51], clinical expertise, and the research team’s knowledge of PROMs, it was hypothesized that KQoL-26 would show strong correlations (r > 0.7) with knee-specific tools (KOOS-PF, KPS, FKIS), as well as with SF-12-PCS, in support of convergent validity. Given that the KQoL-26 contains more physical than mental items, a lower correlation (r < 0.5) with SF-12-MCS was expected. For discriminant validity, weak correlations (r < 0.5) were expected with descriptive variables: age, weight (kg), height (cm), body mass index (BMI), time since the onset of pain (months), Q-angle, and hours of physical activity of the participants; since they were considered non-determinant in the development or severity of the condition [4].

The external validity of the adapted questionnaire was examined to ensure its applicability in different contexts and population groups to guarantee its suitability for those with diverse cultural and demographic characteristics and to confirm the generalizability of the results.

Three measurement properties of reliability were assessed at both the total scale and subscale levels: internal consistency, temporal stability using the test–retest method, and agreement parameters. The latter encompassed the standard error of the mean (SEM), to analyze the precision of our sample mean compared to the population mean; and the smallest detectable change (SDC) to detect meaningful changes beyond random error [52]. The error percentage expressed the SEM as a relative value. Homoscedasticity was also assessed to ensure similar variance in the results.

Discriminant ability was assessed by analyzing floor and ceiling effects, defined as over 15% of participants scoring at the extremes for the total score and for each dimension [53].

Feasibility was assessed through completion time, missing responses, questionnaire completion rate, and participant-reported difficulties. Items with over 5% missing responses were flagged as potentially problematic [54], providing insight into the questionnaire’s usability and clarity.

### 2.5. Statistical Analysis

A sample size calculation was performed with an expected concordance rate of 95% (p = 0.95) and a non-concordance margin of 5% (q = 0.05). A 95% confidence level was applied ($Z_{\frac{\alpha }{2}}=1.96$) with a desired precision of ±4.5% (d = 0.045). A minimum sample size of 91 participants was calculated using the formula:

$$n=\frac{z_{\alpha /2}^{2}\cdot p\cdot q}{d^{2}}=\frac{1.96^{2}\cdot 0.95\cdot 0.05}{0.045^{2}}=90.11≅91$$

Additionally, the COSMIN guidelines [55] and the simulation-based findings of MacCallum et al. [56] were considered, which considered including 100 or more participants optimal in such cases. To account for potential dropouts, the final sample size included a slightly higher number of participants.

Descriptive statistics summarized the demographic and clinical characteristics of the participants. Absolute (N) and relative (%) frequencies were reported for categorical variables, while continuous variables were analyzed based on their distribution. Normality was assessed using the Kolmogorov–Smirnov test for samples of n ≥ 50 and the Shapiro–Wilk test for n < 50. Parametric variables were summarized as mean and standard deviation (SD), whereas nonparametric variables were summarized as median and interquartile range (IQR).

Statistical analyses were performed at the participant and knee level depending on the outcome. Knee-specific instruments (KOOS-PF, KPS, and FKIS) were completed for each affected knee and analyzed independently in bilateral cases, each paired with the corresponding KQoL-26 score. QoL measures (KQoL-26 and SF-12) were analyzed at the participant level.

Factor analysis examined the questionnaire’s structure, beginning with the assessment of sampling adequacy using the Kaiser–Meyer–Olkin (KMO) test and Bartlett’s test of sphericity. A KMO value > 0.7 [57] and a significant Bartlett’s test (*p* < 0.05) were considered appropriate for the analysis. Factor analysis used the principal component extraction method, with Varimax or Promax rotation depending on the approach. Factors were retained based on eigenvalues greater than 1.0 and inspection of the scree plot. The proportion of variance explained was deemed acceptable if it exceeded 50%. Item communalities were also examined and interpreted as very low (<0.30), low (0.30–0.39), acceptable (0.40–0.49), good (0.50–0.69), or very good/excellent (≥0.70) [58]. Factor loadings > 0.4 were considered satisfactory, and in cases where an item loaded onto multiple factors, it was assigned to the factor with the highest correlation, provided the difference between loadings was >0.2 [58].

Construct validity was assessed by interpreting correlation strength based on established guidelines [59]: r > 0.7, strong; 0.7 ≥ r > 0.5, moderate; 0.5 ≥ r > 0.25, weak; and r ≤ 0.25, rare correlation. Either Pearson’s or Spearman’s correlation coefficient was used, depending on data distribution.

Regarding reliability, internal consistency was measured using Cronbach’s α, with values ≥ 0.7 [60,61] considered adequate. Test–retest reliability was assessed using the intraclass correlation coefficient (ICC) and interpreted in the same way as the correlation coefficients described earlier. For agreement, SEM was calculated as SD × √(1 − ICC) [62], and SDC as 1.96 × √2 × SEM [62]. Error percentage was calculated as (SEM/average) × 100; and classified as very good (≤5%), good (>5–10%), doubtful (>10–20%), or inadequate (>20%) [63]. Bland–Altman plots were constructed to assess test–retest agreement of the KQoL-26. Additionally, plots were used to compare differences between KQoL-26 and KOOS-PF, KPS, and FKIS, relative to the mean of KQoL-26 and each respective questionnaire.

To assess the impact of including bilateral knees, a sensitivity analysis was performed by randomly selecting one affected knee per participant.

All statistical analyses were performed by the study authors, with support from an experienced statistician, using IBM SPSS Statistics 28 (SPSS Inc., Chicago, IL, USA). A *p*-value < 0.05 was considered statistically significant.

## 3. Results

The Spanish adaptation of the KQoL-26 is available in Supplementary Materials.

The sample consisted of 104 subjects, of whom 41 had bilateral PFP, making a total of 145 affected knees. Note that analyses were conducted at participant or knee level, depending on the outcome measure (knee-specific outcomes at knee level; QoL measures at participant level). Descriptive data are in Table 1 and Table 2.

### 3.1. Validity

**Figure 1:**
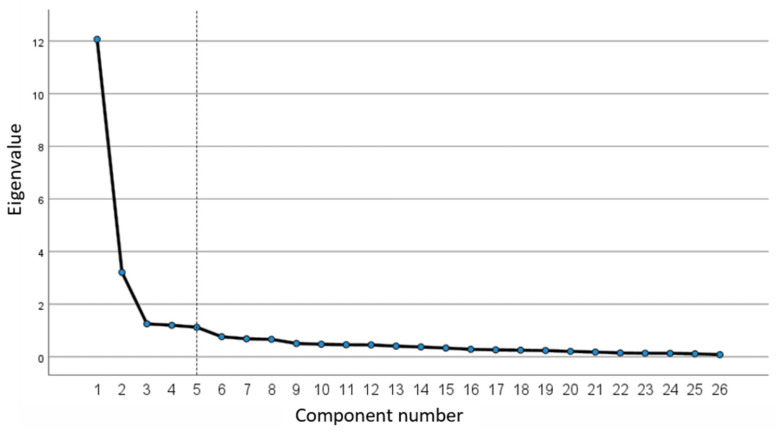
Scree plot of eigenvalues. The dashed vertical line indicates the number of retained components.

The suitability of the data for factor analysis was confirmed by a KMO of 0.915 and a significant Bartlett’s test of 6205.611 (*p* < 0.01). Factor analysis identified 5 factors with eigenvalues > 1.0, a solution supported by the scree plot (Figure 1), and a >50% threshold, collectively explaining 72.5% of the total variance. The factor loadings for each item following Varimax rotation were all > 0.4 (Table 3). Item communalities ranged from 0.520 to 0.840, corresponding to good and very good/excellent levels (Table 4).

The convergent validity analysis showed a strong correlation (r > 0.7) between the total score and each dimension of the KQoL-26 with the rest of the questionnaires, except for the SF-12-MCS. The data are in Table 5.

### 3.2. Reliability

Cronbach’s α and ICC for the overall questionnaire were 0.95. SEM = 3.64, with an associated SDC = 10.08 and an error percentage = 4.63%.

Reliability indices for the total scale and subscales are presented in Table 6.

**Figure 2:**
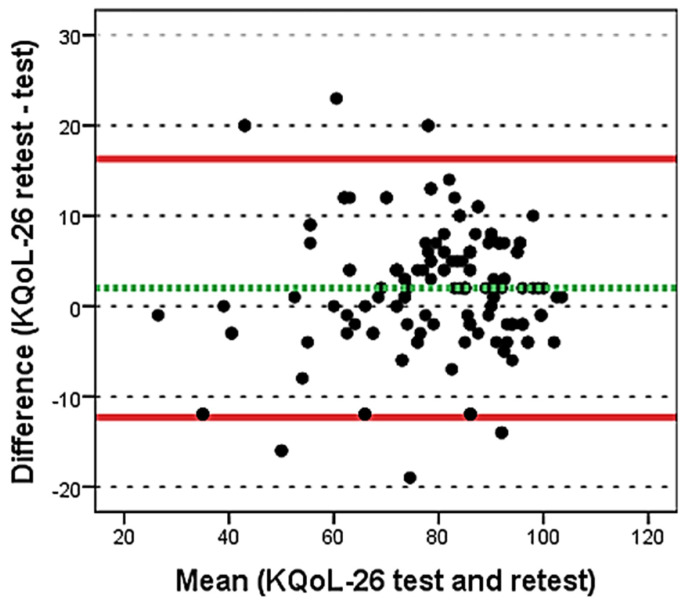
Bland–Altman plot between KQoL-26 test and retest measurements. Limits of agreement: mean difference (green dots line) ± SD × 1.96 (red solid lines) in points for a 100-point scale.

The mean differences between KQoL-26 test and retest measurements were 2.0 (±7.3 SD), with limits of agreement from −12.3 to 16.3, and 93.7% of values within these limits (Figure 2).

**Figure 3:**
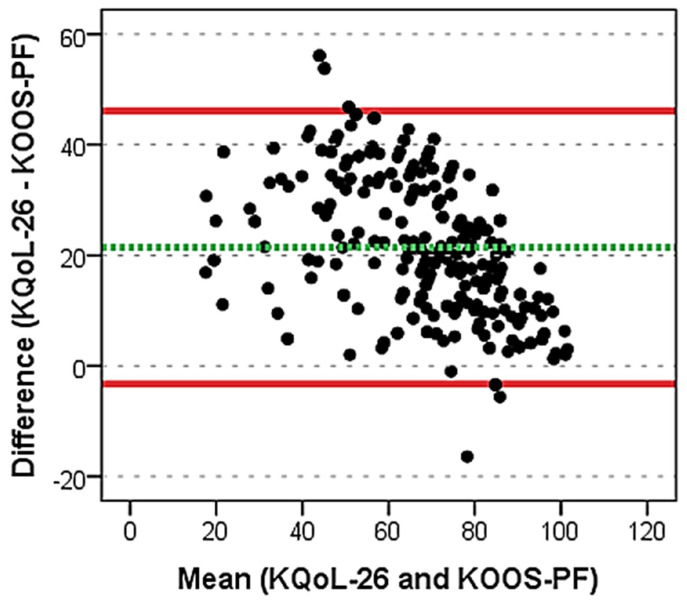
Bland–Altman plot between KQoL-26 and KOOS-PF measurements. Limits of agreement: mean difference (green dots line) ± SD × 1.96 (red solid lines) in points for a 100-point scale.

**Figure 4:**
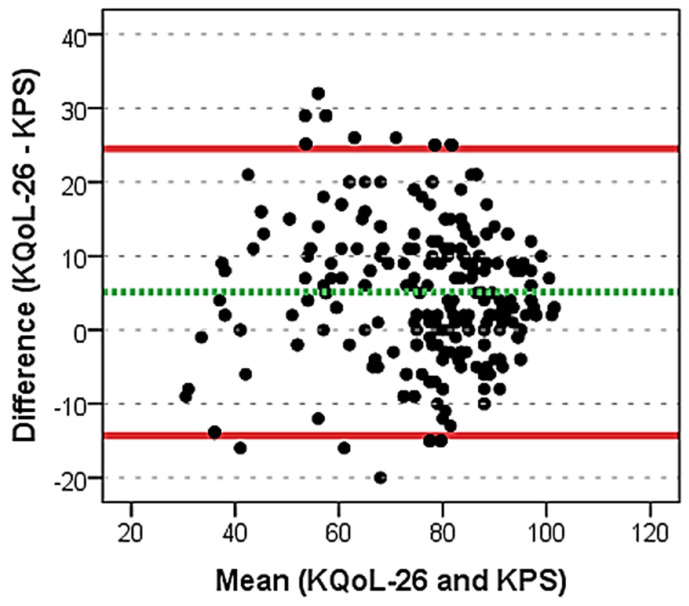
Bland–Altman plot between KQoL-26 and KPS measurements. Limits of agreement: mean difference (green dots line) ± SD × 1.96 (red solid lines) in points for a 100-point scale.

**Figure 5:**
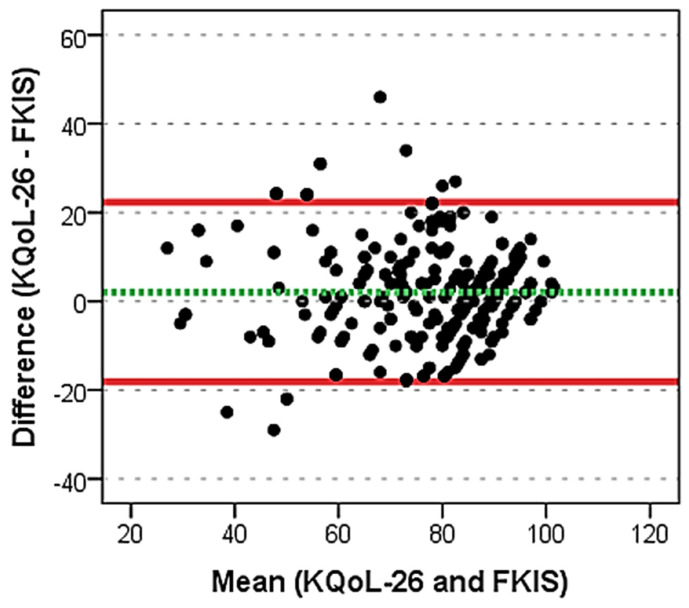
Bland–Altman plot between KQoL-26 and FKIS measurements. Limits of agreement: mean difference (green dots line) ± SD × 1.96 (red solid lines) in points for a 100-point scale.

The mean differences between KQoL-26 and KOOS-PF, KPS, and FKIS were 21.4 (±12.6 SD), 5.1 (±9.9 SD), and 2.1 (±10.3 SD), respectively. The limits of agreement ranged from −3.3 to 46.1 for KOOS-PF, −14.3 to 24.5 for KPS, and −18.1 to 22.3 for FKIS. The Bland–Altman plots (Figure 3, Figure 4 and Figure 5) confirmed that most differences fell within the limits of agreement, with 97.2% in the KOOS-PF plot, 92.1% in the KPS plot, and 95.9% in the FKIS plot, except for a few outliers.

No systematic bias was observed, as the zero line remained within the 95% confidence interval.

### 3.3. Sensitivity Analysis

The sensitivity analysis showed results consistent with the primary analysis, with similar internal consistency (α = 0.949), test–retest reliability (ICC = 0.953), and factor structure (five factors explaining 72.0% of the total variance). Correlations remained within similar ranges.

### 3.4. Discriminant Ability

A total of 2% of participants scored the maximum value, while none scored the minimum value, indicating no floor or ceiling effects. Similarly, none of the dimensions showed floor or ceiling effects.

### 3.5. Feasibility

All participants completed the questionnaire in full, with an average completion time of 278 s (±46 SD). No participants reported difficulties in understanding the items, while seven individuals requested clarification regarding how to respond to items 1–15.

## 4. Discussion

This study translated and cross-culturally adapted the KQoL-26 questionnaire into Spanish, demonstrating robust psychometric properties including strong validity, where a new division into dimensions has been obtained; excellent reliability; as well as adequate discriminant ability. This, together with the success and ease of completion, makes this questionnaire a suitable tool for the assessment of knee-related QoL in the Spanish PFP population.

The translations and back-translations were consistent with the original English version [20]. However, items 4 and 5 required conversion to metric units, since miles are not commonly used in Spain. Moreover, no changes were required following pilot testing, supporting the clarity and cultural adequacy of the adapted version.

The factor structure in our study (5 dimensions) differs from the original validation of the questionnaire (3 dimensions) [20], which initially identified five dimensions, but retained three based on clinical interpretability and Rasch analysis [20]. Our analysis revealed a different five-dimensional structure supported by factor analysis, which was considered clinically reasonable to retain given the excellent KMO (0.915), significant Bartlett (*p* < 0.01), and adequate communalities and factor loadings (>0.4). The five-factor solution should be interpreted cautiously as an exploratory and clinically interpretable structure rather than a confirmed factor model.

The original dimensionality consisted of Physical Functioning, Activity Limitations, and Emotional Functioning. Activity Limitations and Emotional Functioning were preserved in this adaptation, while Physical Functioning (items 1–15) was subdivided into three factors according to knee demand. The Hard physical functioning dimension (items 7, 10–13) implied significant knee flexion (80°/90° or more) under weight-bearing load, which increases the biomechanical demand on the patellofemoral joint [64,65,66] (e.g., kneeling and stair climbing). The Intermediate physical functioning dimension (items 1–4, 8) included weight-bearing activities with less patellofemoral joint demand (<90° flexion) than in the Hard dimension (e.g., lifting, running, walking on uneven ground, walking long distances, and prolonged standing). The Easy physical functioning dimension (items 5, 6, 9, 14, 15) included low-demand tasks such as walking short distances, standing briefly, and basic transfers, and incorporated items 14 and 15 due to possible upper-limb compensation.

This subdivision of the Physical Functioning dimension into levels would allow for more precise detection of clinically significant changes and facilitate rehabilitation planning by identifying affected levels of knee demand and supporting more precise patient monitoring over time. Recent evidence also suggests that PROMs with adequate psychometric properties enhance patient-centered rehabilitation and longitudinal assessment of functional outcomes [67,68]. This aligns with multidimensional assessment approaches in sports population groups [69].

Unlike the original KQoL-26 validation [20] and the Turkish adaptation [70], which analyzed correlations with their individual dimensions, this adaptation considered the total questionnaire score. Furthermore, the number and type of external measures used for comparison were more limited: both the original validation and the Turkish adaptation included three instruments, though only one (Lysholm Score) in the original and two (KOOS and WOMAC) in the Turkish version were knee functional assessment questionnaires. In contrast, this study included four instruments, with three knee-specific questionnaires (KOOS-PF, KPS, and FKIS), providing a broader comparison with knee-focused functional measures.

The three hypotheses established in this study, referring to convergent and discriminant validity, were consistent with the observed results. Construct validity was supported by strong correlations (r > 0.7) for convergent validity, confirming the coherence of the construct measured. However, the correlation with the SF-12-MCS was not significant.

The original KQoL-26 validation [20] reported strong correlations between Lysholm and the dimensions Physical Functioning (r = 0.76) and Activity Limitations (r = 0.76), results that are similar to our correlation with FKIS (r = 0.75). This was expected, as Lysholm was the precursor of FKIS. High correlations were also observed between KQoL-26 and both KOOS-PF (r = 0.82) and KPS (r = 0.79), consistent with previously reported strong correlations between FKIS and KOOS-PF [40], as well as between FKIS and KPS [40,71]. These results align with the Turkish adaptation [70], where the KQoL-26 showed moderate to strong correlations with related constructs.

This study additionally examined correlations with SF-12, showing a stronger association with the PCS (r = 0.75) than with SF-12-MCS (r = 0.05). The pattern is consistent with previous reports of weak associations between SF-12-PCS and SF-12-MCS (r < 0.2) [72,73,74] and with its predominantly physical focus (15 physical items vs. 6 mental items). Additionally, the SF-12 scoring method, where each item contributes to both components with different weights [75], may have influenced the observed difference in correlations. Overall, the low correlation with SF-12-MCS suggests that the questionnaire’s psychosocial items reflect the functional and social limitations of PFP that are specific to the condition rather than general mental health assessed by SF-12-MCS.

Discriminant validity analysis revealed statistically significant but not clinically relevant correlations between KQoL-26 and the descriptive variables, suggesting that KQoL-26 focuses more on PFP-related QoL than on other variables.

The external validity of the adapted questionnaire was supported by the inclusion of a diverse sample, encompassing variations in age, anthropometric characteristics, physical activity levels, professional backgrounds, and geographic areas, including both urban and rural settings. Additionally, data collection was conducted across different seasons to minimize potential biases, particularly in pain perception [76,77,78]. However, sample variability reflects heterogeneity rather than evidence of broad external validity, so generalizability should be limited to similar Spanish individuals with PFP.

The results indicated high reliability. Internal consistency was adequate across dimensions (α = 0.83–0.93), exceeding the adequacy threshold of 0.7 [61], and was comparable to previous versions [20,70]. Similarly, test–retest reliability demonstrated adequate stability over time across dimensions (ICC = 0.77–0.89). While ICC values were slightly lower than those reported in the original validation (ICC = 0.80–0.93) [20] and the Turkish adaptation (ICC = 0.95–0.96) [70], they remained within acceptable ranges, supporting the reliability of the adapted questionnaire.

Regarding agreement, SEM (3.64) and error percentage (4.63%) reflected very good measurement precision, while SDC (10.08) indicated that changes above this threshold reflect true change. Bland–Altman plots confirmed strong consistency between KQoL-26 and the other questionnaires, with >90% of cases within the limits of agreement. The test–retest Bland–Altman analysis further supported good agreement over time, with 93.7% of observations within the limits of agreement.

Sensitivity analysis confirmed that including bilateral knees did not affect the psychometric properties of the KQoL-26, supporting the robustness of the findings.

The absence of floor and ceiling effects in the KQoL-26 adaptation demonstrates its ability to capture the full spectrum of knee-related QoL.

Feasibility was supported by high completion rates and a shorter completion time (22 s less than the original validation [20], which lasted 5 min), suggesting that the Spanish adaptation is efficient. Regarding clarity, seven participants expressed uncertainty when answering items 1–15, where responses were based on the degree of limitation in performing the tasks (e.g., “Very limited”, “A little limited”, etc.). Participants were unsure whether to account for the presence of pain in their responses, i.e., whether being able to perform a task despite pain was considered a limitation. Pain, as a key symptom of the pathology, was clarified as a relevant limitation, even if tasks could still be completed. This suggests a slight potential ambiguity in the interpretation of the response scale, reported by only 7 of 104 participants, which may warrant clarification in future applications of the instrument. Despite this, the Spanish KQoL-26 was well received, with all participants finding the questions relevant to their health status and daily activities.

Regarding the study limitations, as this was a cross-cultural adaptation without longitudinal follow-up or intervention, neither changes over time nor the responsiveness of the instrument could be assessed, as this was beyond the scope of the present design. Therefore, the authors propose prospective clinical trials applying the current version of the questionnaire in the Spanish population to subsequently assess its responsiveness. Additionally, despite sample diversity, the monocentric and geographically limited sample restricts generalizability to similar population groups. It does not support direct extension to other Spanish-speaking regions, such as Latin America, without further validation. Content validity was based on qualitative expert review, without calculating a quantitative content validity index. The use of convenience sampling may also limit representativeness. Moreover, no subgroup analyses were performed between unilateral and bilateral cases, which may limit the assessment of potential differences between these groups. Furthermore, SF-12 data were limited to a subset of participants due to a protocol amendment, which may affect the robustness of analyses involving these variables. Finally, a confirmatory factor analysis was not performed due to the use of the same sample for both adaptation and exploratory analysis; therefore, future studies should confirm the factor structure in independent samples using confirmatory factor analysis or Rasch analysis.

Regarding strengths, this study conducted a comprehensive and rigorous adaptation of the KQoL-26, addressing relevant and necessary psychometric aspects: validity (structural, construct, and external), reliability (internal consistency, temporal stability, and agreement), discriminant ability, and feasibility. The robust results obtained support its potential use in other population groups, not only due to language but also considering factors such as gender, age, sociocultural context, clinical condition, etc., given the heterogeneity of the sample employed. In addition, the availability of a culturally adapted Spanish version may facilitate implementation of standardized PROMs and improve longitudinal monitoring of rehabilitation outcomes. This aligns with recent recommendations encouraging the broader use of cross-culturally adapted lower-extremity PROMs in clinical settings [79,80]. Furthermore, the findings of this study encourage future cross-cultural adaptations into other languages to further expand its use and support a more holistic approach that addresses both physical and psychosocial aspects to better understand and manage pain and its clinical consequences.

## 5. Conclusions

A cross-cultural adaptation of the KQoL-26 questionnaire into Spanish was conducted, along with an assessment of its psychometric properties in PFP. The adaptation demonstrated excellent measurement properties, including validity, reliability, discriminant ability, and feasibility. As a result, the newly validated version appears to be a suitable tool for assessing QoL in Spanish-speaking users with PFP. It may support both clinical decision-making and research by providing a comprehensive assessment of patient-reported outcomes. However, further multicentric and longitudinal studies are needed to confirm its generalizability, responsiveness, ability to detect clinical change, and minimal clinically important difference.

## Figures and Tables

**Table 1:** Descriptive characteristics of the participants.

Participants			Men (n = 34)	Women (n = 70)	Total (n = 104)
Age (years)		$\overset{-}{x}$ (SD)	34.4 (12.7)	34.2 (13.1)	34.3 (13.0)
		Med (IRQ)	30 (21–47)	28 (22–48)	30 (22–47)
Weight (kg)		$\overset{-}{x}$ (SD)	85.1 (12.9)	67.3 (12.0)	72.9 (14.8)
		Med (IRQ)	83 (75–95)	65 (58–75)	73 (61–82)
Height (cm)		$\overset{-}{x}$ (SD)	180 (7)	165 (6)	170 (10)
		Med (IRQ)	181 (178–184)	165 (160–170)	169 (163–178)
BMI		$\overset{-}{x}$ (SD)	26.2 (3.5)	24.7 (4.7)	25.2 (4.4)
		Med (IRQ)	24.9 (23.4–29.0)	23.4 (20.8–27.0)	24.5 (21.9–27.7)
Impairment	Unilateral	N (%)	22 (21.2)	41(39.4)	63 (60.6)
	Bilateral	N (%)	12 (11.5)	29 (27.9)	41 (39.4)
Impaired limb ^a^	Dominant	N (%)	13 (21)	21 (33)	34 (54)
	Non-dominant	N (%)	9 (14)	20 (32)	29 (46)
Physical activity (hours per week)		$\overset{-}{x}$ (SD)	6.5 (3.4)	5.1 (4.0)	5.5 (3.9)
		Med (IRQ)	6 (4–8)	5 (2–7)	5 (3–7)
Region	Urban	N (%)	18 (17)	46 (44)	64 (61)
	Rural	N (%)	16 (15)	24 (23)	40 (38)
Occupation	Management/Professional	N (%)	7 (7)	19 (18)	26 (25)
	Service	N (%)	9 (9)	24 (23)	33 (32)
	Office work	N (%)	7 (7)	6 (6)	13 (13)
	Student	N (%)	11 (11)	21 (20)	32 (31)
KQoL-26		$\overset{-}{x}$ (SD)	83.6 (10.7)	76.3 (17.6)	78.6 (16.1)
		Med (IRQ)	85 (76–91)	79.5 (66–90)	82 (70.0–90.3)
SF-12	SF-12-PCS	$\overset{-}{x}$ (SD)	49.8 (6.3)	47.5 (9.7)	47.9 (9.2)
		Med (IRQ)	51.1 (44.1–54.1)	49.5 (41.2–55.7)	50 (42.7–55.9)
	SF-12-MCS	$\overset{-}{x}$ (SD)	43.7 (13.1)	49.2 (8.3)	48.2 (9.5)
		Med (IRQ)	47.3 (31–55.9)	50.5 (42.0–56.1)	50.5 (41.2–55.9)

Abbreviations: $\overset{-}{x}$, mean; SD, standard deviation; Med, median; IQR, interquartile range; BMI, body mass index; KQoL-26, Knee Quality of Life 26-item questionnaire; SF-12, 12-item Short Form health survey; SF-12-PCS, 12-item Short Form health survey Physical Component Summary; SF-12-MCS, 12-item Short Form health survey Mental Component Summary. ^a^ Only unilateral affected subjects were considered. Notes: Both measurement moments were considered for the statistics of the questionnaire. The SF-12 statistics were based on a subsample (n = 25) due to protocol expansion during data collection. Questionnaire score ranges: 0–100 (optimum).

**Table 2:** Descriptive characteristics of the participants’ affected knees.

Knees		Men (n = 46)	Women (n = 99)	Total (n = 145)
Time with pain (months)	$\overset{-}{x}$ (SD)	90.9 (73.7)	77.8 (77.2)	81.9 (76.1)
	Med (IRQ)	72 (33–138)	60 (18–108)	72 (24–120)
Affected knees Q-angle (degrees)	$\overset{-}{x}$ (SD)	15.9 (3.7)	20.4 (4.5)	19.0 (4.7)
	Med (IRQ)	16 (14–18)	20 (17–24)	18 (16.0–22.5)
KOOS-PF ^a^	$\overset{-}{x}$ (SD)	63.4 (20.1)	54.3 (22.5)	57.2 (22.1)
	Med (IRQ)	63.6 (50.0–80.7)	56.8 (35.8–72.7)	61.4 (40.9–72.7)
KPS ^a^	$\overset{-}{x}$ (SD)	79.1 (11.9)	71.0 (19.0)	73.5 (17.5)
	Med (IRQ)	80 (73–87)	77 (55–88)	78 (62–87)
FKIS ^a^	$\overset{-}{x}$ (SD)	80.9 (12.8)	74.6 (16.7)	76.6 (15.8)
	Med (IRQ)	85 (69.3–90.0)	80 (65–89)	81.5 (68–89)

Abbreviations: $\overset{-}{x}$, mean; SD, standard deviation; Med, median; IQR, interquartile range; KOOS-PF, Knee Injury and Osteoarthritis Outcome Score for Patellofemoral Pain and osteoarthritis; KPS, Kujala Patellofemoral Score; FKIS, Fulkerson Knee Instability Scale. ^a^ Participants with bilateral PFP provided responses for each knee individually where relevant. Note: both measurement moments were considered for the statistics of the questionnaire. Questionnaire score ranges: 0–100 (optimum).

**Table 3:** Rotated component matrix.

Items	Components				
	1	2	3	4	5
22	0.88				
23	0.86				
24	0.86				
26	0.85				
25	0.80				
21	0.72				
18	0.34	0.72			
17	0.31	0.72			
20		0.63	0.46		
19	0.36	0.60	0.34		
16	0.39	0.56	0.35		
2			0.78		
4			0.78		
3			0.77	0.32	
8		0.49	0.51		.33
1		0.51	0.51		
12			0.33	0.81	
13				0.78	
11		0.36		0.75	
10		0.47		0.68	
7		0.32	0.37	0.42	0.33
6					0.81
9		0.31			0.70
5			0.52		0.60
14		0.57			0.52
15		0.49	0.33		0.40

Extraction method: main component analysis. Rotation method: Varimax with Kaiser normalization. Note: The rotation has converged after 6 iterations.

**Table 4:** Item communalities.

Communalities		
	Initial	Extraction
KQoL-26.1	1.00	0.68
KQoL-26.2	1.00	0.69
KQoL-26.3	1.00	0.79
KQoL-26.4	1.00	0.80
KQoL-26.5	1.00	0.73
KQoL-26.6	1.00	0.76
KQoL-26.7	1.00	0.52
KQoL-26.8	1.00	0.66
KQoL-26.9	1.00	0.73
KQoL-26.10	1.00	0.78
KQoL-26.11	1.00	0.82
KQoL-26.12	1.00	0.81
KQoL-26.13	1.00	0.81
KQoL-26.14	1.00	0.69
KQoL-26.15	1.00	0.54
KQoL-26.16	1.00	0.66
KQoL-26.17	1.00	0.67
KQoL-26.18	1.00	0.76
KQoL-26.19	1.00	0.66
KQoL-26.20	1.00	0.75
KQoL-26.21	1.00	0.60
KQoL-26.22	1.00	0.84
KQoL-26.23	1.00	0.82
KQoL-26.24	1.00	0.76
KQoL-26.25	1.00	0.72
KQoL-26.26	1.00	0.81

Extraction method: main component analysis.

**Table 5:** Correlation among KQoL-26 and KOOS-PF, KPS, FKIS, and SF-12.

r	KOOS-PF	KPS	FKIS	SF-12-PCS	SF-12-MCS
KQoL-26	0.82 (*p* < 0.001)	0.79 (*p* < 0.001)	0.75 (*p* < 0.001)	0.75 (*p* < 0.001)	0.05 (*p* = 0.695)

Abbreviation: r, correlation coefficient; KQoL-26, Knee Quality of Life 26-item questionnaire; KOOS-PF, Knee injury and Osteoarthritis Outcome Score for Patellofemoral pain and osteoarthritis; KPS, Kujala Patellofemoral Score; FKIS, Fulkerson Knee Instability Scale; SF-12-PCS, 12-item Short Form health survey Physical Component Summary; SF-12-MCS, 12-item Short Form health survey Mental Component Summary. Notes: all coefficients were analyzed using Spearman’s Rho. Regarding discriminant validity analysis, weight and amount of physical activity showed non-significant correlations with KQoL-26. Age, BMI, and time with pain were weakly correlated with the questionnaire (0.5 ≥ r > 0.25). Height and Q-angle exhibited a rare correlation (r < 0.25).

**Table 6:** Internal consistency, test–retest reliability, and measurement error of the total scale and subscales.

	α	ICC	SEM	SDC
Total scale	0.95 (0.94–0.96) *	0.95 (0.94–0.96) *	3.64 (3.40–4.01) *	10.08 (9.42–11.13) *
Dimension 1	0.88	0.89	1.56	4.32
Dimension 2	0.83	0.77	1.11	3.09
Dimension 3	0.89	0.84	1.59	4.41
Dimension 4	0.89	0.84	1.57	4.35
Dimension 5	0.93	0.84	2.15	5.97

* 95% CI reported only for total scale estimates.

## Data Availability

The data that support the findings of the current study are available from the corresponding author on reasonable request.

## Supplementary Materials

The following supporting information can be downloaded at: https://www.mdpi.com/article/10.3390/jcm15135102/s1, Questionnaire S1: Spanish version of “Knee Quality of Life 26-item Questionnaire”.

## Abbreviations

The following abbreviations are used in this manuscript:

PFP	Patellofemoral Pain
QoL	Quality of Life
KPS	Kujala Patellofemoral Score
FKIS	Fulkerson Knee Instability Scale
KOOS-PF	Knee injury and Osteoarthritis Outcome Score for Patellofemoral pain and osteoarthritis
KQoL-26	Knee Quality of Life 26-item questionnaire
COSMIN	Consensus-based Standards for the selection of health Measurement Instruments
PROMs	Patient-reported outcome measures
GRRAS	Guidelines for Reporting Reliability and Agreement Studies
SF-12	12-item Short Form health survey
KOOS	Knee injury and Osteoarthritis Outcome Score
SF-12-PCS	12-item Short Form health survey Physical Component Summary
SF-12-MCS	12-item Short Form health survey Mental Component Summary
BMI	Body Mass Index
SEM	Standard Error of the Mean
SDC	Smallest Detectable Change
KMO	Kaiser–Meyer–Olkin
ICC	Intraclass Correlation Coefficient
