# Supplementary material for: Cross-Cultural Adaptation and Validation of Knee Quality of Life 26-Item Questionnaire into Spanish in Patellofemoral Pain
Source: J Clin Med. 2026 Jun 30;15(13):5102. doi: 10.3390/jcm15135102 (PMC13362749; doi:10.3390/jcm15135102)
Supplement: Supplementary file 1 [file jcm-15-05102-s001.zip › jcm-4362231-supplementary.pdf]

Supplementary Material: Questionnaire S1.

Spanish version of "Knee Quality of Life 26-item Questionnaire"

Versión Española de "Knee Quality of Life 26-item Questionnaire"

Nombre y apellidos: ____

Las siguientes preguntas se refieren a los problemas que le ha causado su rodilla. Por favor, responda a cada pregunta con una cruz. Si no está seguro de cómo responder a una pregunta, de la mejor respuesta que pueda.

Estas preguntas se refieren a las actividades que puede realizar durante un día normal. ¿Su rodilla le limita actualmente en estas actividades? Si es así, ¿en qué medida?

Por favor, marque únicamente una casilla de cada línea

	**Totalmente limitado / Incapaz**	**Muy limitado**	**Limitación moderada**	**Poco limitado**	**Sin limitación**
1. Coger a un niño u **objetos pesados** como bolsas de la compra o pequeños muebles	☐	☐	☐	☐	☐
2. Correr	☐	☐	☐	☐	☐
3. Caminar en **terreno irregular**	☐	☐	☐	☐	☐
4. Caminar **8 kilómetros**	☐	☐	☐	☐	☐
5. Caminar **1.5 kilómetros**	☐	☐	☐	☐	☐
6. Cruzar la carretera	☐	☐	☐	☐	☐
7. Arrodillarse	☐	☐	☐	☐	☐
8. Estar de pie durante **una hora**	☐	☐	☐	☐	☐
9. Estar de pie durante **cinco minutos**	☐	☐	☐	☐	☐
10. **Subir varios tramos** de escalones	☐	☐	☐	☐	☐
11. **Subir un tramo** de escalones	☐	☐	☐	☐	☐
12. **Bajar varios tramos** de escalones	☐	☐	☐	☐	☐
13. **Bajar un tramo** de escalones	☐	☐	☐	☐	☐

14. Sentarse	☐	☐	☐	☐	☐
15. Acostarse o salir de la cama	☐	☐	☐	☐	☐

Ahora nos gustaría preguntarle sobre las últimas 4 semanas. Por favor, responda a cada pregunta con una cruz. Si no está seguro de cómo responder a una pregunta, por favor, de la mejor respuesta que pueda.

16. Durante las **últimas 4 semanas**, ¿en qué grado sintió que su rodilla le frenaba en sus actividades diarias?

Por favor, marque una casilla

☐	☐	☐	☐	☐
**En todo momento**	**La mayoría del tiempo**	**En algunos momentos**	**En pocos momentos**	**En ningún momento**

17. Durante las **últimas 4 semanas**, ¿en qué grado le ha impedido su rodilla viajar en coche o en transporte público (incluyendo autobuses y trenes)?

Por favor, marque una casilla

☐	☐	☐	☐	☐
**En todo momento**	**La mayoría del tiempo**	**En algunos momentos**	**En pocos momentos**	**En ningún momento**

18. Durante las **últimas 4 semanas**, ¿cuánto tiempo ha interferido su rodilla en su trabajo habitual (incluyendo el trabajo fuera de casa y las tareas domésticas)?

Por favor, marque una casilla

☐	☐	☐	☐	☐
**En todo momento**	**La mayoría del tiempo**	**En algunos momentos**	**En pocos momentos**	**En ningún momento**

19. Durante las **últimas 4 semanas**, ¿en qué grado ha interferido su rodilla en las actividades o relaciones sociales (como visitar a los amigos, salir a comer o ir al cine o al teatro)?

Por favor, marque una casilla

☐	☐	☐	☐	☐
**En todo momento**	**La mayoría del tiempo**	**En algunos momentos**	**En pocos momentos**	**En ningún momento**

20. Durante las **últimas 4 semanas**, ¿cuánto tiempo ha interferido su rodilla a la hora de hacer recados como hacer la compra o ir a la oficina de correos o al banco?

Por favor, marque una casilla

☐	☐	☐	☐	☐
**En todo**	**La mayoría del**	**En algunos**	**En pocos**	**En ningún**

**momento**	**tiempo**	**momentos**	**momentos**	**momento**

21. Durante las **últimas 4 semanas**, ¿cuánto tiempo ha pasado pensando en su rodilla?

Por favor, marque una casilla

☐	☐	☐	☐	☐
**En todo momento**	**La mayoría del tiempo**	**En algunos momentos**	**En pocos momentos**	**En ningún momento**

22. Durante las **últimas 4 semanas**, ¿cuántas veces se ha sentido enfadado o molesto a causa de su rodilla?

Por favor, marque una casilla

☐	☐	☐	☐	☐
**En todo momento**	**La mayoría del tiempo**	**En algunos momentos**	**En pocos momentos**	**En ningún momento**

23. Durante las **últimas 4 semanas**, ¿cuántas veces se ha sentido desanimado o triste a causa de su rodilla?

Por favor, marque una casilla

☐	☐	☐	☐	☐
**En todo momento**	**La mayoría del tiempo**	**En algunos momentos**	**En pocos momentos**	**En ningún momento**

24. Durante las **últimas 4 semanas**, ¿cuánto tiempo le ha preocupado que su rodilla empeore?

Por favor, marque una casilla

☐	☐	☐	☐	☐
**En todo momento**	**La mayoría del tiempo**	**En algunos momentos**	**En pocos momentos**	**En ningún momento**

25. Durante las **últimas 4 semanas**, ¿cuánto tiempo se ha sentido de mal humor por su rodilla?

Por favor, marque una casilla

☐	☐	☐	☐	☐
**En todo momento**	**La mayoría del tiempo**	**En algunos momentos**	**En pocos momentos**	**En ningún momento**

26. Durante las **últimas 4 semanas**, ¿cuánto tiempo se ha sentido frustrado por su rodilla?

Por favor, marque una casilla

☐	☐	☐	☐	☐
**En todo momento**	**La mayoría del tiempo**	**En algunos momentos**	**En pocos momentos**	**En ningún momento**
